# Assessment of Free Radical Scavenging Potential and Oxidative DNA Damage Preventive Activity of *Trachyspermum ammi* L. (Carom) and *Foeniculum vulgare* Mill. (Fennel) Seed Extracts

**DOI:** 10.1155/2014/582767

**Published:** 2014-07-23

**Authors:** Nandini Goswami, Sreemoyee Chatterjee

**Affiliations:** Department of Biotechnology, The IIS University, Gurukul Marg, SFS, Mansarovar, Jaipur, Rajasthan 302020, India

## Abstract

Oxidation of biomolecules such as carbohydrates, proteins, lipids, and nucleic acids results in generation of free radicals in an organism which is the major cause of onset of various degenerative diseases. Antioxidants scavenge these free radicals, thereby protecting the cell from damage. The present study was designed to examine the free radical scavenging potential and oxidative DNA damage preventive activity of traditionally used spices *Trachyspermum ammi* L. (carom) and *Foeniculum vulgare* Mill. (fennel). The aqueous, methanolic, and acetonic extracts of *T. ammi* and *F. vulgare* seeds were prepared using soxhlet extraction assembly and subjected to qualitative and quantitative estimation of phytochemical constituents. Free radical scavenging potential was investigated using standard methods, namely, DPPH radical scavenging assay and ferric reducing antioxidant power assay along with the protection against oxidative DNA damage. The results stated that acetonic seed extracts (AAcSE and FAcSE) of both the spices possessed comparatively high amount of total phenolics whereas methanolic seed extracts (AMSE and FMSE) were found to have highest amount of total flavonoids. At 1 mg/mL concentration, highest DPPH radical scavenging activity was shown by FMSE (96.2%), AAcSE was recorded with highest FRAP value (2270.27 ± 0.005 *μ*mol/L), and all the seed extracts have been shown to mitigate the damage induced by Fenton reaction on calf thymus DNA. Therefore, the study suggests that *T. ammi* and *F. vulgare* seed extracts could contribute as a highly significant bioresource of antioxidants to be used in our day-to-day life and in food and pharmaceutical industry.

## 1. Introduction

Recent era is being focused on screening of medicinal plants/seeds for their phytoconstituents which have proven to be possible agents that can be used as drugs and various pharmaceutical products. Several naturally occurring phytoconstituents such as tocopherols, carotenoids, ascorbates, polyphenolics, and terpenoids from plants have been analysed and used as alternative therapeutic agents to cure various diseases caused by oxidative stress which thereby generate free radicals [1–3]. Free radicals which have one or more unpaired electron stabilize themselves by electron pairing with biological macromolecules such as lipids, proteins, and DNA causing the damage, along with lipid peroxidation in healthy human cells resulting in onset of several diseases such as cardiovascular diseases, inflammatory diseases, atherosclerosis, ageing, and cancer [4–6]. Hence, antioxidant supplements in daily diet need to be incorporated when protective mechanism of human cells is disrupted.

Spices have been used since antiquity as flavor enhancers and to preserve food due to the presence of antioxidant phytochemicals [7]. In recent years, due to their incredible effects, much attention is driven towards the exploration of bioactive compounds, extracted from medicinal plants/spices to minimize the use of synthetic drugs because of associated adverse effects. Medicinal plants/spices are also in high demand due to their several beneficial applications and thus highly preferred in functional food and biopharmaceutical industries because of consumer choices [8].

*T. ammi* and* F. vulgare* belonging to Apiaceae family are the most common spices, known for their highly aromatic nature and flavour in culinary and traditional applications. Earlier Mediterranean Region and Southern Europe were native places for growth of* F. vulgare* but at present the plant is widely cultivated in the tropical and temperate regions of the world. Various pharmacological activities of* F. vulgare* such as antioxidant [9], hepatoprotective [10], antimicrobial [11], oestrogenic [12], acaricidal [13], antihirsutism [14], antidiabetic [15], anti-inflammatory [16], and antithrombotic [17] ones have been reported in literature.

*T. ammi* seeds are cultivated in Afghanistan, India, Iran, Iraq, and Pakistan.* T. ammi *seeds are reported to possess antimicrobial [18], antioxidant [19], hepatoprotective [20], nematicidal [21], antihelminthic [22], and gastroprotective activities [23].

Present study was designed to evaluate and report the qualitative and quantitative determination of phytochemical compounds and* in vitro* antioxidant activity of various extracts of* T. ammi *and* F. vulgare* seeds along with their protective effects on calf thymus DNA damage induced by Fenton reaction.

## 2. Materials and Methods

### 2.1. Collection of Plant Sample

*Trachyspermum ammi *L. (carom) and* Foeniculum vulgare* Mill. (fennel) seeds were procured from the National Research Centre for Seeds and Spices (NRCSS), Tabiji farm, Ajmer, Rajasthan.

Authentication no.:

 
*Trachyspermum ammi* L.—AA1
 
*Foeniculum vulgare* Mill.—AF1.

### 2.2. Chemicals

All the chemicals and solvents used in the assays were of high quality and analytical grade. Most of the chemicals and solvents purchased from Himedia, India.

### 2.3. Preparation of Extracts from Seeds of Carom and Fennel

Soxhlet extraction assembly was used for the preparation of methanolic and acetonic seed extracts. As per the protocol defined by Javed et al., 2012, 50 g of dried and powdered seeds of both carom and fennel was mixed with 250 mL methanol/acetone and continuous extraction was done for 5 to 6 hours [24]. For preparation of aqueous seed extract, about 60 g of the powdered seeds was mixed in 1 L of distilled water and placed on a mechanical shaker for 48 hours. The extract was filtered using a Whatman no. 1 filter paper [25]. The resulting slurry of all the extracts obtained was dried in oven and final powdered extract was stored at 4°C. The various extracts were coined as per the convenience, for example,* T. ammi,* that is, ajwain aqueous seed extract (AASE), methanolic seed extract (AMSE), and acetonic seed extract (AAcSE), and that of* F. vulgare*, fennel aqueous seed extract (FASE), fennel methanolic seed extract (FMSE), and fennel acetonic seed extract (FAcSE).

### 2.4. Phytochemical Analysis

The powdered seed extracts were evaluated for qualitative determination of major phytoconstituents, that is, carbohydrates, alkaloids, phenols, flavonoids, tannins, and phytosterols using different methods as listed in Table 1. Besides quantitative determination of phenols and flavonoids was also carried out.

### 2.5. Determination of Total Phenolic Content

Total phenolic content in the extracts was determined using modified Folin-Ciocalteu method [35]. 0.5 mL of the extract was mixed with 5 mL Folin-Ciocalteu reagent (Himedia India) (previously diluted with water at 1 : 10 v/v) and 4 mL (75 g/L) of sodium carbonate (Himedia India). The tubes were vortexed for 15 sec and incubated at 40°C for 30 min in water-bath. Absorbance was measured at 765 nm using the Hewlett Packard UV-VS spectrophotometer. Samples of extracts were evaluated at a final concentration of 10 mg/mL. Total phenolic content was expressed as mg/g tannic acid equivalent, using the following equation based on the calibration curve [36]:

(1) $$\begin{array}{c} y=0.1216x,\\ R^{2}=0.9365, \end{array}$$

where *x* = absorbance and *y* = tannic acid equivalent (mg/g).

### 2.6. Determination of Total Flavonoid Content

Total flavonoid content in the plant extracts was determined according to the method of Ordoñez et al. [37]. To 0.5 mL of sample, 0.5 mL of 2% AlCl_3_-ethanol solution was added. After incubation at room temperature for one hour, absorbance was measured at 420 nm. Appearance of yellow color indicated the presence of flavonoids. Extracted samples were evaluated for flavonoids at concentration of 10 mg/mL. Total flavonoid content was expressed in terms of quercetin (mg/g) using the following equation based on the calibration curve [36]:

(2) $$\begin{array}{c} y=0.0255x,\\ R^{2}=0.9812, \end{array}$$

where *x* = absorbance and *y* = quercetin equivalent (mg/g).

### 2.7. *In Vitro* Antioxidant Assay

A stock solution of concentration 10 mg/mL was maintained for all the extracts of* T. ammi *and* F. vulgare. *The various extracts were diluted in respective solvents in the range 0.2–1 mg/mL for various antioxidant assays and reducing ability testing. Antioxidant power of each assay was compared with that of the standard antioxidant compounds.

#### 2.7.1. 2,2-Diphenyl-1-picrylhydrazyl (DPPH) Radical Scavenging Activity

The effect of* T. ammi* and* F. vulgare* seed extracts on DPPH radical was estimated using the method of Liyana-Pathiranan and Shahidi [38]. A solution of 0.135 mM DPPH (Himedia India) in methanol was prepared and 1.0 mL of this solution was mixed with 1.0 mL of extract. The reaction mixture was then incubated in the dark at room temperature for 30 min. The absorbance of the mixture was measured spectrophotometrically at 517 nm. Butylhydroxytoluene (BHT) was used as standard reference.

The ability of the extracts to scavenge DPPH radical was calculated by the following equation:

(3) $$\begin{array}{c} \text{Inhibition }\left(\%\right)=\left[\frac{\left({\text{Abs}}_{\text{control}}-{\text{Abs}}_{\text{sample}}\right)}{\left({\text{Abs}}_{\text{control}}\right)}\right]\times 100, \end{array}$$

where

(4) $$\begin{array}{c} {\text{Abs}}_{\text{control}}=\text{absorbance of DPPH radical}+\text{methanol}\\ {\text{Abs}}_{\text{sample}}=\text{absorbance}\;\text{of}\;\text{DPPH}\;\text{radical}+\text{sample}. \end{array}$$

#### 2.7.2. Ferric Reducing Antioxidant Potential

The determination of reducing ability of* T. ammi* and* F. vulgare* seed extracts was done according to the method of Benzie and Strain [39]. The FRAP reagent constituents were 300 mM acetate buffer (3.1 g sodium acetate trihydrate and 16 mL glacial acetic acid, pH 3.6), 10 mM TPTZ (2,4,6-tripyridyl-*s*-triazine) solution in 40 mM HCl, and 20 mM ferric chloride hexahydrate solution. The working solution was always freshly prepared by mixing 25 mL acetate buffer, 2.5 mL TPTZ, and 2.5 mL ferric chloride hexahydrate. The temperature of the solution was maintained at 37°C prior to use. Plant extracts/standard (100 *μ*L) were allowed to react with 3 mL of FRAP solution and 300 *μ*L distilled water for 4 min in the dark condition. Absorbance of the colored product (ferrous tripyridyltriazine complex) was taken at 593 nm. Results were expressed in *μ*M Fe (II)/mL of extract and compared with that of BHT.

### 2.8. Protective Effect of Seed Extracts against H_2_O_2_-Induced DNA Damage

To study the protective effects of all the seed extracts of* T. ammi* and* F. vulgare* against DNA damage induced by Fenton reaction, the reaction was conducted in a microcentrifuge tube at a total volume of 15 *μ*L containing 0.5 *μ*g of calf thymus DNA, 3 *μ*L of 50 mM phosphate buffer (pH 7.4), 3 *μ*L of 2 mM FeSO_4_, and 2 *μ*L of the seed extracts at highest (1 mg/mL) concentrations. Then, 4 *μ*L of 30% H_2_O_2_ was added, and the mixture was incubated at 37°C for 1 h [40]. Finally, the mixture was subjected to 1% agarose gel electrophoresis.

### 2.9. Statistical Analysis

The experimental results were expressed as mean ± standard deviation (SD) of three replicates. Statistical significance was determined using Student's* t*-test.* P* value < 0.05 was considered as significant.

## 3. Results and Discussion

*T. ammi *and* F. vulgare *are known for their characteristic aromatic smell and taste and have widespread use as a traditional medicine. The seeds are used as preservatives, as flavoring agents, in medicine, and for the preparation of essential oil in perfumery [41]. Various pharmacological properties of these spices have also been explored. The therapeutic benefits of the spices are usually contributed to their antioxidant properties. The biochemical investigation reported that* T. ammi *and* F. vulgare* seeds constitute bioactive compounds such as anethole and thymol, respectively, which significantly contribute to the antioxidant potential [9, 19]. The phytochemical investigation of* T. ammi *and* F. vulgare *seed extracts as summarized in Table 2 revealed the presence of various phytoconstituents such as carbohydrates, alkaloids, phenols, phytosterols, tannins, and flavonoids which are known to possess medicinal as well as physiological activities. Alkaloids are mixed groups or compounds which mostly contain nitrogen bearing molecules that make them pharmacologically active. Flavonoids possess antioxidant elements and ensure healthy circulation.

Polyphenols act as antioxidants and protect cells and body chemicals against damage caused by free radicals and reactive atoms. They can also block the action of enzymes that cancer needs for growth and deactivate substances that promote the growth of cancers. Quantitative estimation proved that all the extracts of* T. ammi* and* F. vulgare *seeds possess significant amount of total phenolics and flavonoids (Table 3); however, acetonic seed extract of both* T. ammi* and* F. vulgare *was comparatively rich in phenolic (0.432 ± 0.9 mg/g tannic acid equivalent in AAcSE and 0.364 ± 1.1 mg/g tannic acid equivalent in FAcSE) compounds. Methanolic seed extracts of* T. ammi* and* F. vulgare *seeds showed the highest amount of flavonoid content, that is, 0.0496 ± 0.49 mg/g quercetin equivalent in AMSE and 0.0449 ± 0.96 mg/g quercetin equivalent in FMSE. Total phenolic content were expressed as mg/g tannic acid equivalent and total flavonoid content was expressed in terms of quercetin (mg/g). Several studies have shown that polyphenolic contents present in herbal plants/spices were responsible for decrease in oxidative stress [42]. Presence of such phytoconstituents in these spices makes them suitable as pharmaceutical product and as potential free radical scavengers that could to an extent provide protection against degenerative and chronic diseases.

Different free radical generating systems were used in this study to assess the free radical scavenging and reducing properties of the aqueous (AASE and FASE), methanolic (AMSE, FMSE), and acetonic (AAcSE, FAcSE) seed extracts. The observed differential scavenging activities of the seed extracts against various systems may be attributed to the different mechanisms of the radical antioxidant reactions in the different assays. Hagerman et al. [43] have reported that the high molecular weight phenolics (tannins) have more abilities to quench free radicals (ABTS^•+^) and their effectiveness depends on the molecular weight, the number of aromatic rings, and nature of hydroxyl groups substitution than the specific functional groups.

**Figure 1:**
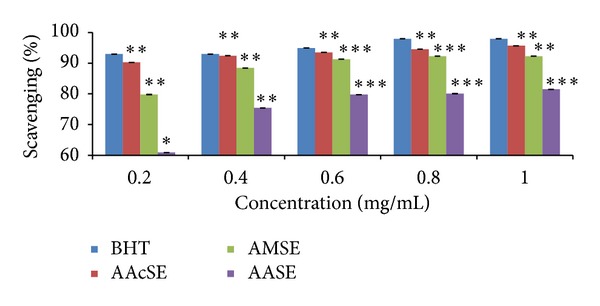
DPPH radical scavenging activity of* Trachyspermum ammi* L. seed extracts (AASE: ajwain aqueous seed extract, AMSE: ajwain methanolic seed extract, and AAcSE: ajwain acetonic seed extract) compared with BHT (butylated hydroxytoluene) as standard antioxidant at different concentrations (0.2–1 mg/mL) in terms of free radical inhibition percentage. Statistical significance was determined using Student's* t*-test. *P* value < 0.05 was considered as significant (** = significant and *** = highly significant).

**Figure 2:**
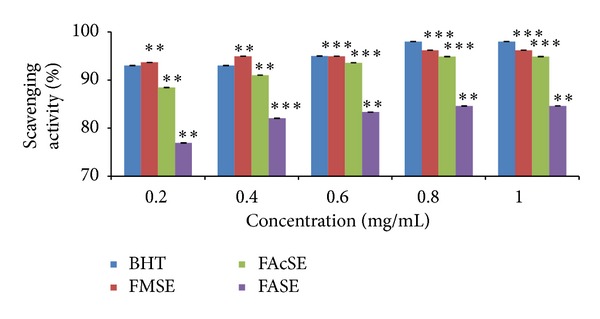
DPPH radical scavenging activity of* Foeniculum vulgare* Mill. seed extracts (FASE: fennel aqueous seed extract, FMSE: fennel methanolic seed extract, and FAcSE: fennel acetonic seed extract) compared with BHT (butylated hydroxytoluene) as standard antioxidant at different concentrations (0.2–1 mg/mL) in terms of free radical inhibition percentage. Statistical significance was determined using Student's* t*-test.* P* value < 0.05 was considered as significant (**= significant and ***= highly significant).

**Figure 3:**
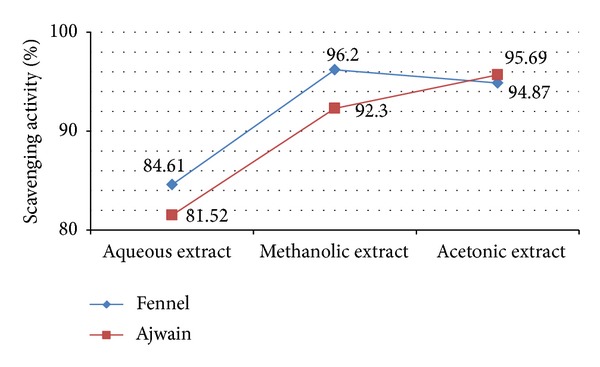
Comparative DPPH radical scavenging activity of all the seed extracts of* Trachyspermum ammi* L. and* Foeniculum vulgare* Mill. at concentration 1 mg/mL.

DPPH is a stable, light sensitive, nitrogen-centered free radical which produces violet color in methanol solution. With the addition of* F. vulgare* and* T. ammi* seed extracts, it was reduced to a yellow colored product diphenylpicryl hydrazine in a dose dependent manner and also resulted in decreased absorbance. The degree of discoloration indicated the scavenging potential of the antioxidant compounds/extracts in terms of hydrogen donating ability [44, 45]. From the study conducted, all the extracts of* T. ammi* and* F. vulgare* seeds revealed their hydrogen donating ability. Figures 1 and 2 represent the DPPH radical scavenging activity of* T. ammi* and* F. vulgare* seed extracts, respectively, at various concentrations compared with BHT (98%) in terms of inhibition percentage in which acetonic seed extract exhibited (AAcSE with 95.69% and FMSE with 96.2% scavenging activity) comparatively highest inhibition percentage, at 1 mg/mL concentration.* T. ammi* and* F. vulgare* seed extracts in terms of DPPH radical scavenging activity were ranked as FASE < FAcSE < FMSE and AASE < AMSE < AAcSE (Figure 3).

**Figure 4:**
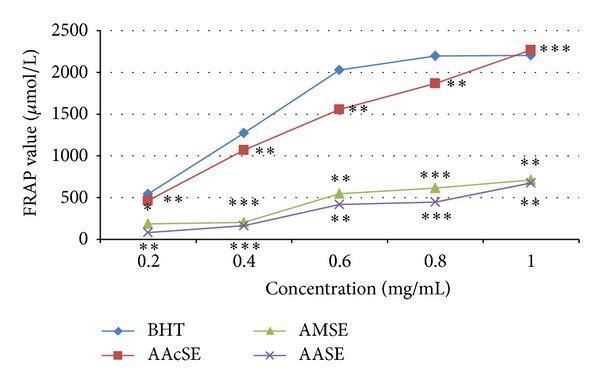
Ferric reducing antioxidant power of* Trachyspermum ammi* L. seed extracts (AASE: ajwain aqueous seed extract, AMSE: ajwain methanolic seed extract, and AAcSE: ajwain acetonic seed extract) compared with BHT (butylated hydroxytoluene) as standard antioxidant at different concentrations (0.2–1 mg/mL) in terms of FRAP value expressed in *µ*mol/L. Statistical significance was determined using Student's* t*-test.* P* value < 0.05 was considered as significant (**= significant and ***= highly significant).

**Figure 5:**
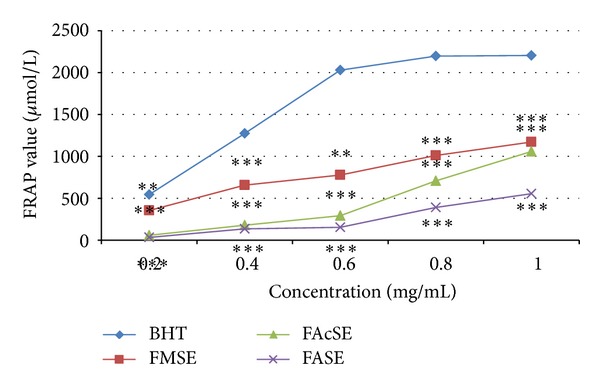
Ferric reducing antioxidant power of* Foeniculum vulgare* Mill. seed extracts (FASE: fennel aqueous seed extract, FMSE: fennel methanolic seed extract, and FAcSE: fennel acetonic seed extract) compared with BHT (Butylated hydroxytoluene) as standard antioxidant at different concentrations (0.2–1 mg/mL) in terms of FRAP value expressed in *µ*mol/L. Statistical significance was determined using Student's* t*-test.* P* value < 0.05 was considered as significant (**= significant and ***= highly significant).

**Figure 6:**
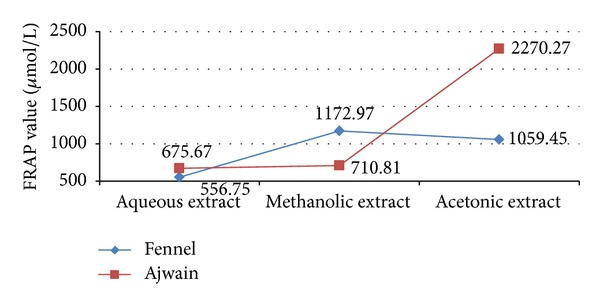
Comparative free radical antioxidant potential of* Trachyspermum ammi* L. and* Foeniculum vulgare* Mill. seed extracts at concentration 1 mg/mL in terms of FRAP value expressed in *µ*mol/L.

In FRAP assay, the antioxidants present in the sample were considered reductants in a redox-linked colorimetric reaction [46]. An antioxidant capable of donating a single electron to the ferric- TPTZ (Fe (III)-TPTZ) complex reduces this complex into the blue ferrous-TPTZ (Fe (II)-TPTZ) complex which absorbs strongly at 593 nm. Antioxidant compounds which act as reducing agent exert their effect by donating hydrogen atom to ferric complex and thus break the radical chain reaction. The higher the absorbance is, the higher the antioxidant activity is, which is indicated by the high FRAP value. Figures 4 and 5 represent the FRAP values of* T. ammi* and* F. vulgare* seed extracts, respectively, at various concentrations and compared with BHT in which the AAcSE was recorded with highest FRAP value 2270.27 ± 0.005 *µ*mol/L and FMSE exhibited 1172.97 ± 0.005 *µ*mol/L FRAP value at 1 mg/mL concentration.* F. vulgare* and* T. ammi* seed extracts were ranked as FASE < FAcSE < FMSE and AASE < AMSE < AAcSE, respectively, in terms of ferric reducing antioxidant potential (Figure 6).

The diversity in radical scavenging shown in these assays may also be due to factors like stereo selectivity of the radicals or the differential solubility that may be justified in case of crude extracts, which contain a variety of antioxidants.

**Figure 7:**
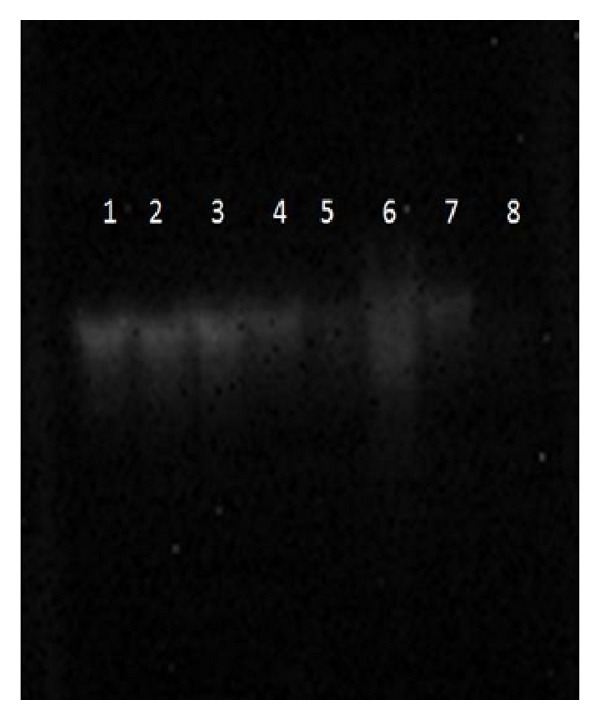
Protective effect of* T. ammi *(AASE, AMSE, and AAcSE) and* F. vulgare* (FASE, FMSE, and FAcSE) seed extracts at 1 mg/mL concentrationon oxidative damage to calf thymus DNA. Lane 1 : 0.5 *μ*g native calf thymus DNA + 50 mM phosphate buffer, lane 2: 0.5 *μ*g DNA + 2 mM FeSO_4_ + 30% H_2_O_2_ + 50 mM phosphate buffer + AAcSE (1 mg/mL), lane 3: 0.5 *μ*g DNA + 2 mM FeSO_4_ + 30% H_2_O_2_ + 50 mM phosphate buffer + AMSE (1 mg/mL), lane 4: 0.5 *μ*g DNA + 2 mM FeSO_4_ + 30% H_2_O_2_ + 50 mM phosphate buffer + AASE (1 mg/mL), lane 5: 0.5 *μ*g DNA + 2 mM FeSO_4_ + 30% H_2_O_2_ + 50 mM phosphate buffer + FAcSE (1 mg/mL), lane 6: 0.5 *μ*g DNA + 2 mM FeSO_4_ + 30% H_2_O_2_ + 50 mM phosphate buffer + FMSE (1 mg/mL), lane 7: 0.5 *μ*g DNA + 2 mM FeSO_4_ + 30% H_2_O_2_ + 50 mM phosphate buffer + FASE (1 mg/mL), and lane 8: 0.5 *μ*g DNA + 2 mM FeSO_4_ + 30% H_2_O_2_ + 50 mM phosphate buffer.

Protective effect of* T. ammi* and* F. vulgare* seed extracts at concentration 1 mg/mL against H_2_O_2_-induced DNA damage in calf thymus DNA is displayed in Figure 7. Fenton reaction generates hydroxyl radicals which are found to induce DNA strand breaks in calf thymus DNA. H_2_O_2_ in the presence of ferrous sulfate leads to high DNA damage (Lane 8).* T. ammi *(AASE, AMSE, and AAcSE) and* F. vulgare* (FASE, FMSE, and FAcSE) seed extracts at 1 mg/mL concentration showed slight protection against DNA damage induced by hydroxyl radicals as compared to control (lane 1) in calf thymus DNA (Lane 2–7); however, the lower concentrations of seed extracts (0.2–0.8 mg/mL) were not able to show any visible protection against DNA damage. Again our results indicate that ajwain acetonic seed extract (AAcSE) and fennel methanolic seed extract (FMSE) showed better protection against DNA damage (lane 8) as compared to other seed extracts of* T. ammi* and* F. vulgare*.

## 4. Conclusion

*Trachyspermum ammi* L. and* Foeniculum vulgare* Mill. are used as traditional medicine since long time. They are flavourful spices with high aromatic odor and culinary and medicinal uses. They have been best known for their various pharmacological properties such as antioxidant, hepatoprotective, anticancer, antifungal, and antibacterial activity. The present study was conducted for qualitative and quantitative determination of phytochemical compounds and to screen the free radical scavenging activity of two commonly used spices, that is,* Trachyspermum ammi *L. (carom) and* Foeniculum vulgare* Mill. (fennel). In assaying the DPPH radical scavenging activity and ferric reducing antioxidant potential, it was concluded that all the seed extracts of* T. ammi* and* F. vulgare* could be used as efficient antioxidant agents comparable to commercially used antioxidant, BHT (butylated hydroxytoluene). The seed extracts have also been shown to mitigate the oxidative damage in calf thymus DNA. These plant extracts can be considered as significant source of natural antioxidants that can withstand the deleterious effects of many diseases such as such as cancer, atherosclerosis, diabetes, inflammation, and ageing.

## Figures and Tables

**Table 1:** Qualitative phytochemical analysis of *Trachyspermum ammi* L. and *Foeniculum vulgare* Mill. seed extracts.

S. number	Phytoconstituents	Tests
1	Alkaloids [26–28]	Mayer's test
		Wagner's test
		Hager's test

2	Carbohydrates [29]	Molisch's test
		Barfoed's test

3	Saponins [30]	Frothing test

4	Phytosterols [31]	Liebermann-Burchard's test
		Salkowski's test

5	Phenols [32]	Ferric chloride test

6	Tannins [26]	Gelatin test

7	Flavonoids [33, 34]	Alkaline reagent test
		Shinoda test

8	Glycosides [26]	Legal's test
		Keller-Kiliani test

9	Fixed oils and fats [30]	Spot test
		Saponification test

**Table 2:** Qualitative phytochemical analysis of *Trachyspermum ammi* L. and *Foeniculum vulgare* Mill. seed extracts.

S. number	Phytoconstituents	Tests	Seed extracts					
			AASE	AMSE	AAcSE	FASE	FMSE	FAcSE
1	Alkaloids [26–28]	Mayer's test	+	+	+	+	+	+
		Wagner's test	+	+	+	+	+	+
		Hager's test	+	+	+	+	+	+

2	Carbohydrates [29]	Molisch's test	+	+	+	+	+	+
		Barfoed's test	+	+	+	+	+	+

3	Saponins [30]	Frothing test	−	−	−	−	−	−

4	Phytosterols [31]	Liebermann-Burchard's test	+	+	+	+	+	+
		Salkowski's test	+	+	+	+	+	+

5	Phenols [32]	Ferric chloride test	+	+	+	+	+	+

6	Tannins [26]	Gelatin test	+	+	+	+	+	+

7	Flavonoids [33, 34]	Alkaline reagent test	+	+	+	+	+	+
		Shinoda test	+	+	+	+	+	+

8	Glycosides [26]	Legal's test	−	−	−	−	−	−
		Keller-Kiliani test	−	−	−	−	−	−

9	Fixed oils and fats [30]	Spot test	−	−	−	−	−	−
		Saponification test	−	−	−	−	−	−

(AASE: ajwain aqueous seed extract, AMSE: ajwain methanolic seed extract, AAcSE: ajwain acetonic seed extract, FASE: fennel aqueous seed extract, FMSE: fennel methanolic seed extract, FAcSE: fennel acetonic seed extract, + = present, − = absent).

**Table 3:** Quantification of total phenolic content and total flavonoid content in *Trachyspermum ammi* L. and *Foeniculum vulgare* Mill. seed extracts.

Seed extracts	Total phenolic content (mg/g tannic acid equivalent)	Total flavonoid content (mg/g quercetin equivalent)
AASE	0.199 ± 1.3	0.038 ± 0.78
AMSE	0.210 ± 0.83	0.049 ± 0.99
AAcSE	0.432 ± 0.9	0.036 ± 0.78
FASE	0.184 ± 1.2	0.042 ± 0.6
FMSE	0.277 ± 2.3	0.044 ± 0.96
FAcSE	0.364 ± 1.1	0.038 ± 0.84

(AASE: ajwain aqueous seed extract, AMSE: ajwain methanolic seed extract, AAcSE: ajwain acetonic seed extract, FASE: fennel aqueous seed extract, FMSE: fennel methanolic seed extract, FAcSE: fennel acetonic seed extract). The values are expressed in mean ± S.D.
